# Clinical Experience using Osimertinib in Patients with Recurrent Malignant Gliomas Containing *EGFR* Alterations

**DOI:** 10.26502/jcsct.5079114

**Published:** 2021-04-29

**Authors:** Marin Abousaud, Naqeeb M Faroqui, Glenn Lesser, Roy E Strowd, Shakti H Ramkissoon, Madan Kwatra, Kristin S Houston, Fang-Chi Hsu, Annette Carter, Robin Petro, Alisha T DeTroye

**Affiliations:** 1 Department of Pharmacy, Emory Healthcare, Atlanta, GA, USA; 2 Department of General Surgery, Wellstar Atlanta Medical Center, Atlanta, GA, USA; 3 Wake Forest Baptist Health Comprehensive Cancer Center, Department of Internal Medicine, Section on Hematology and Oncology, Winston-Salem, NC, USA; 4 Wake Forest Baptist Health Comprehensive Cancer Center, Department of Pathology, Winston-Salem, NC, USA; 5 Foundation Medicine, Morrisville, NC, USA; 6 Department of Anesthesiology, Duke University Medical Center, Durham, NC, USA; 7 Department of Biostatistics and Data Science, Division of Public Health Sciences, Wake Forest School of Medicine, Winston-Salem, NC, USA

**Keywords:** Osimertinib, *EGFR*, *EGFR vIII*, Tyrosine kinase inhibitor, Glioma, Glioblastoma, GBM, Astrocytoma, Targeted therapy, Precision oncology

## Abstract

**Background:**

*EGFR* alterations are commonly observed in malignant gliomas (MG). Osimertinib, an irreversible EGFR-tyrosine kinase inhibitor, effectively penetrates the blood brain barrier and achieves therapeutic concentrations in brain tissue.

**Materials and Methods:**

This retrospective chart review identified six patients with recurrent MG and *EGFR* alterations who received osimertinib.

**Results:**

Four patients were assessed for response. One patient had a partial response, two patients achieved stable disease and one was refractory. One patient with an *EGFR vIII* rearrangement remained on treatment for 236 days and a second patient with an *EGFR vIII* mutation remained on treatment for 294 days and continued on treatment at the time of analysis. Thrombocytopenia occurred in two patients, one patient developed grade 1 diarrhea and pneumonia, and another patient developed grade 1 mucositis.

**Conclusion:**

Osimertinib had a tolerable safety profile in this heavily pretreated brain tumor population. Osimertinib may benefit select patients with recurrent MG containing *EGFR* alterations.

## 1. Introduction

Gliomas are the most prevalent malignant primary brain tumor in adults, accounting for approximately 30% of all central nervous system tumors and 80% of all malignant brain tumors [1]. Maximal safe resection followed by radiation therapy with concurrent and/or adjuvant chemotherapy is considered the standard of care for malignant gliomas (MGs) [2]. As with other cancers, there has been a need for the development of effective targeted agents for gliomas, since most patients will progress and require further treatment. Finding effective treatments for these primary malignant brain tumors has been challenging due to their chemo- and radio-resistant properties, paucity of targetable genomic aberrations, and the presence of the blood brain barrier (BBB) which potentially limits the entry of systemically administered therapeutics.

In recent decades, the epidermal growth factor receptor (EGFR) signaling pathway has garnered a great deal of attention due to its role in cancer pathogenesis and the availability of novel therapies that specifically and effectively target this pathway [3, 4]. Several studies have suggested a correlation between *EGFR* alterations and glioma tumor growth, survival, invasion, and angiogenesis [5, 6]. *EGFR* alterations are more commonly observed in patients with glioblastomas (GBM) in comparison to low-grade gliomas [4]. Many *EGFR* variants have been identified in gliomas, including amplification, overexpression, insertion-deletion (indel), point mutations, rearrangements, and other aberrations [7]. Based on The Cancer Genome Atlas (TCGA) program, the four major *EGFR* alterations identified in GBM are: 1. *EGFR* with a large deletion in the extracellular domain (*EGFR vIII*); 2. *EGFR* with kinase domain duplication (*EGFR-KDD*); 3. wildtype (wt) *EGFR* amplification; and 4. *EGFR* fused with *SEPT-14* (*EGFR-SEPT14)* [8, 9]. In patients who overexpress *EGFR*, 50–60% of them can also have the *EGFR vIII* mutation. *EGFR vIII* is characterized by a gene rearrangement that deletes exons 27 which results in ligand-independent (constitutive) phosphorylation and activation of the *EGFR* receptor and signaling pathway [4, 5].

*EGFR*-tyrosine kinase inhibitors (EGFR-TKIs), such as gefitinib, erlotinib, afatinib, and dacomitinib, have been studied in *EGFR*-altered gliomas but have yielded minimal to no clinical benefit and short durations of response [10–14]. Unlike the other *EGFR-TKIs*, osimertinib (AZD9291), a third-generation, irreversible *EGFR-TKI* commonly used to treat *EGFR*-mutant lung cancer, is able to effectively penetrate the BBB and achieve therapeutic concentrations in brain tissue, making it an attractive option for gliomas with *EGFR* alterations [15, 16]. Additionally, osimertinib has been shown to be a more tolerable agent in comparison to the other first- and second-generation EGFR-TKIs [16]. There have been in vitro and in vivo studies supporting the preclinical activity of osimertinib in *EGFR* expressing GBM, particularly with GBM that express *EGFR vIII* [9, 17]. Due to the scarcity of studies describing the impact of osimertinib on gliomas in humans, we report our clinical experience of using osimertinib in patients with gliomas containing *EGFR* alterations.

## 2. Materials and Methods

This was an observational, single-center, retrospective chart review that was approved by the Wake Forest University Health Sciences Institutional Review Board (Approval Number: IRB00065716). A report was generated from the EPIC electronic medical record for patients who received osimertinib for gliomas with *EGFR* alterations within the Wake Forest Baptist Comprehensive Cancer Center (WFBCCC) system. Data was extracted from medical records and stored in REDCap (Research Electronic Data Capture), which is HIPAA compliant and includes audit trails to ensure patient confidentiality.

To be eligible for the study, patients were required to have histologically confirmed glioma with known *EGFR* alterations identified by next generation sequencing (NGS) (Foundation Medicine, Inc., Cambridge, MA, USA) and have received at least one dose of osimertinib. As previously described, DNA extracted from formalin-fixed, paraffin-embedded tumor tissues were assayed by adaptor ligation hybrid capture-based NGS [18, 19]. Sequencing data was analyzed for genomic alterations, including short variant alterations (base substitutions, insertions, and deletions), copy number alterations (focal amplifications and homozygous deletions), and select gene fusions or rearrangements [18, 19].

Patients received osimertinib 80 mg by mouth once daily and continued treatment until disease progression, the development of unacceptable side effects, medical complications, or death. For the majority of patients, osimertinib was not covered by insurance due to its off-label use in MGs. Through the Precision Oncology program at WFBCCC, team members were able to facilitate the process of obtaining osimertinib for off-label indication for these patients.

### 2.1 Endpoints

Study aims were to describe our experience using osimertinib in patients with gliomas with *EGFR* alterations and to assess the safety and tolerability of osimertinib in this patient population. Other outcome measures included best response, time to disease progression, and toxicity while on osimertinib.

### 2.2 Definitions

Best response was assessed on brain MRIs using the Response Assessment in Neuro-Oncology (RANO) criteria (complete response [CR], partial response [PR], stable disease [SD], progression of disease [PD]/refractory) [20]. Time to progression was defined as time from date of treatment initiation to date of disease progression or death from any cause. Best response and disease progression were determined by the treating physician’s and radiologist’s interpretation of the imaging and clinical course documented in the patient’s medical record. Toxicity was evaluated according to the National Cancer Institute Common Terminology Criteria for Adverse Events (NCI-CTCAE) version 5.0 [21].

### 2.3 Statistical analysis

The sample size was small. Descriptive statistics were utilized for demographic data and all outcome measures. Response rates were summarized by count and frequency.

## 3. Results

From January 1, 2018 to February 4, 2021, six patients were identified for inclusion in the study. The median age at diagnosis was 56.5 years (range: 46–74) and they were primarily males (83.3%). Four patients had a pathologic diagnosis of GBM while two patients were diagnosed with anaplastic astrocytomas. Three patients had a gross total resection during their first surgery, two had stereotactic biopsies, and one had a subtotal resection. The median number of prior surgeries, recurrences, and prior regimens before osimertinib treatment was 2 (range: 2–3), 2 (range: 1–2), and 2.5 (range: 2–3), respectively. Three of the six patients were MGMT unmethylated, two patients were methylated, and one patient’s MGMT status was unknown. The median Karnofsky performance status (KPS) score at the start of osimertinib therapy was 70.

All patients had previously received radiation plus temozolomide prior to osimertinib. The majority of patients began osimertinib as a third line of treatment and three of the patients were taking it concurrently with another agent: bevacizumab (two patients) or temozolomide (one patient). The median length of time from patient consent to initiation of osimertinib was 40 days. All of the patients received glucocorticoids concurrently with osimertinib. Two patients had a potential drug-drug interaction with osimertinib; however, this did not lead to any osimertinib dose adjustments or toxicities. Both patients were on carbamazepine; one patient had only received one dose of osimertinib while on carbamazepine and the other patient took carbamazepine approximately two times per week as needed for trigeminal neuralgia. Based on the discretion of the treating provider, a dose adjustment for osimertinib was not warranted since carbamazepine was taken sparingly.

**Figure 1:**
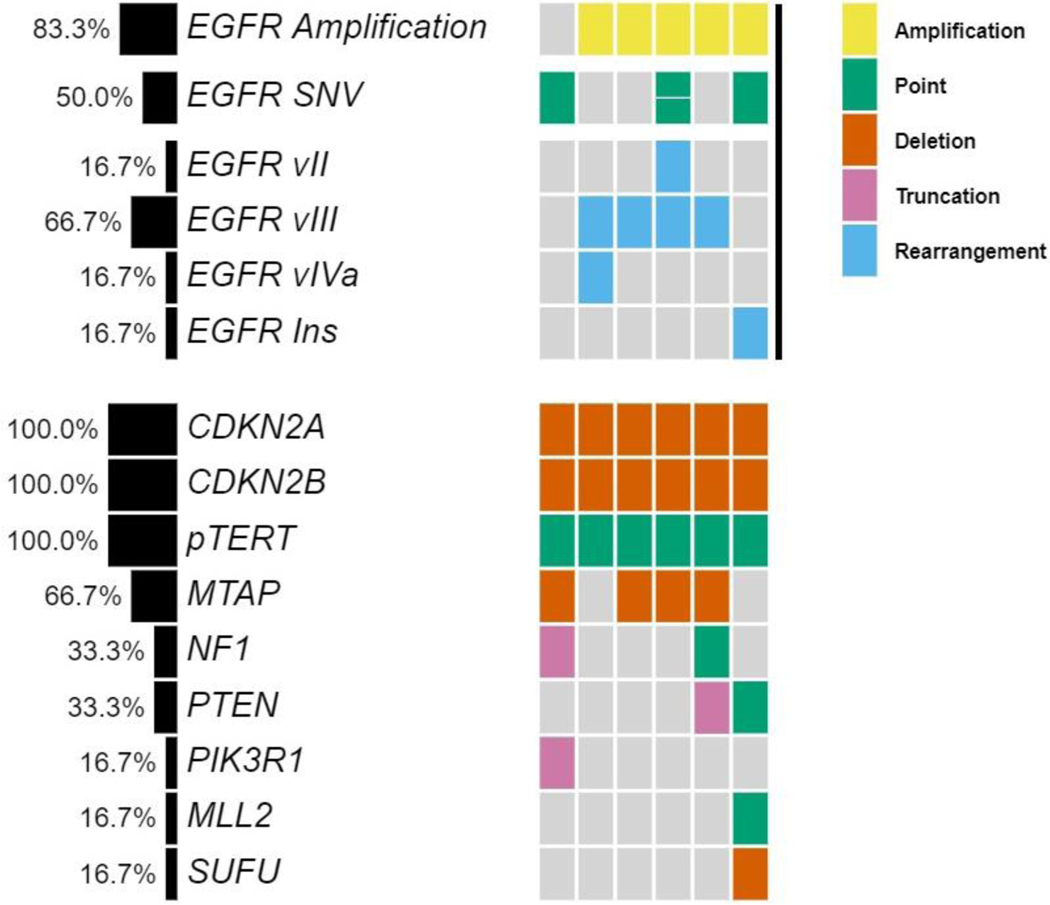
Co-mutation plot of six patients treated with osimertinib highlights heterogeneity of *EGFR* alterations and genomic profiles.

Genomic profiling revealed that all patients were *IDH1/2* wild type. *EGFR* amplification was detected in five patients, four of which showed co-occurring *EGFR vIII* rearrangements characterized by intragenic deletion of exons 2–7. Other structural rearrangements identified included one patient with *EGFR vII* (deletion of exons 1415) and one with *EGFR vIVa* (deletion of exons 25–27) (Figure 1 and Table 1).

**Figure 2.**
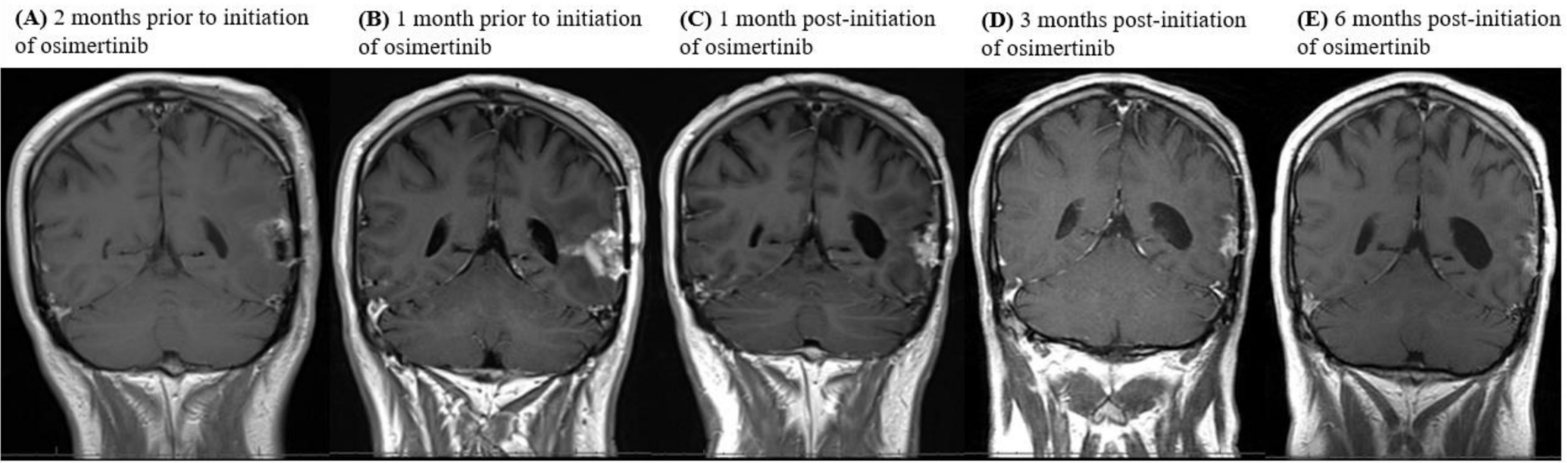
**MRI images detailing pre- and post-treatment with osimertinib.** **(A)** Post-operative imaging after undergoing a left temporal craniotomy for recurrent glioblastoma (WHO Grade IV), 2 months prior to initiation of osimertinib. **(B)** 1 month post-operative imaging prior to patient beginning any therapy. Patient was stalled on treatment with daily temozolomide 50 mg/m2 and Optune following this scan. Osimertinib was initiated 1 month later. Temozolomide was held upon initiation of osimertinib. **(C)** 1 month post-initiation of osimertinib. Patient resumed daily temozolomide 50 mg/m2 and continued on osimertinib and Optune. **(D)** 3 months post-initiation of osimertinib therapy in combination with daily low-dose temozolomide and Optune. **(E)** 6 months post-initiation of osimertinib therapy in combination with daily low-dose temozolomide and Optune. The patient continues on treatment with osimertinib, daily temozolomide 50 mg/m2, and Optune after 294 days. Imaging continues to show stable disease.

**Figure 3.**
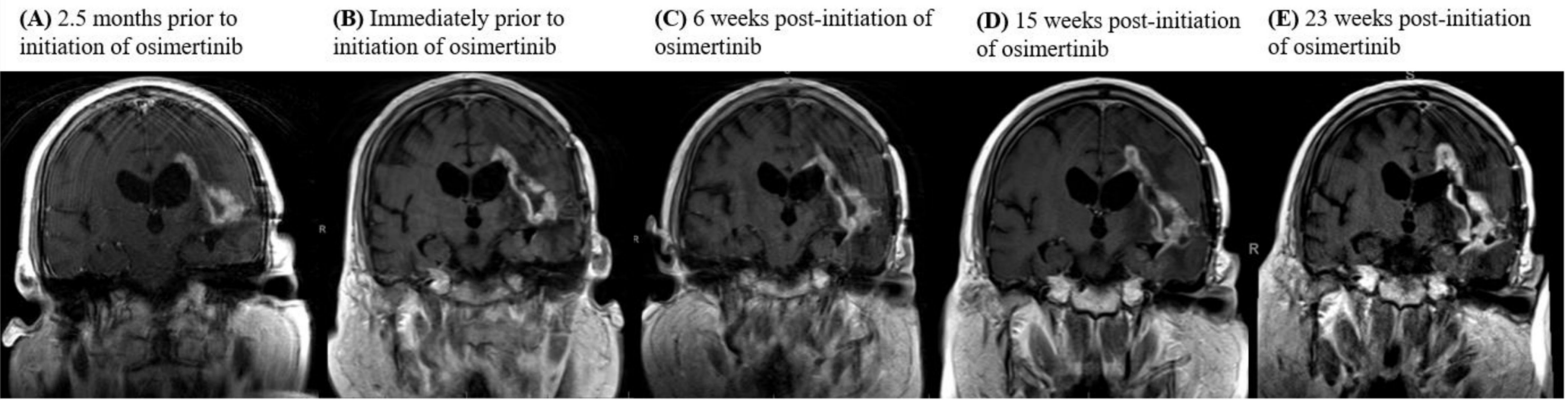
**MRI images detailing pre- and post-treatment with osimertinib.** **(A)** 2.5 months prior to starting osimertinib therapy, the patient was off all therapy. **(B)** The patienťs left temporal lobe lesion progressed while he was off treatment. Osimertinib was initiated. **(C)** After 6 weeks of osimertinib therapy, the lesion remained stable. **(C)** After 15 weeks of osimertinib therapy, the lesion remained stable. **(D)** After 23 weeks of osimertinib therapy, the lesion remained stable. The patient eventually developed progression of disease after 236 days on osimertinib therapy and osimertinib was discontinued.

Consistent with their classification as MGs, all tumors showed homozygous deletion of *CDKN2A/B* and *TERT* promoter mutations. A subset of tumors also harbored mutations such as G598V, R108K, T263P and A289V, as *EGFR* single nucleotide variations (SNV) or point well as co-occurring mutations (Figure 1 and Table 1). Of the six patients, only four could be assessed for response. Out of the four patients, one patient achieved partial response, two patients achieved a best response of stable disease while on osimertinib and one patient was refractory to treatment. The patient with PR has an *EGFR vIII* mutation and continues on osimertinib after 294 days in conjunction with temozolomide and Optune. One patient with an *EGFR vIII* mutation and SD on osimertinib remained on treatment for 236 days prior to progression. The other patient progressed after 77 days of treatment. The patient who was on osimertinib for 77 days had a transient improvement on imaging, which may also have reflected an increase in dexamethasone dosing. Neuroimaging findings for the patient with PR and longest SD on osimertinib are shown in Figures 2 and 3.

In terms of the safety profile of osimertinib, four of six patients experienced an adverse event. One patient had grade 2 thrombocytopenia, one patient had grade 3 thrombocytopenia (was on concurrent bevacizumab therapy), one patient had grade 1 mucositis and one patient had grade 1 diarrhea and pneumonia. Three patients experienced mild myelosuppression that was felt to be unrelated to osimertinib. No rash was noted for any of the patients. Most patients did not have baseline and follow up electrocardiogram (EKGs) or echocardiograms (ECHOs) performed to monitor for QTc prolongation and cardiomyopathy/decreased left ventricular ejection fraction, respectively. For the one patient whom had a baseline and follow up EKG obtained, no QTc prolongation was observed while on osimertinib. A summary of patient demographics with their corresponding responses and tolerability can be found in Table 2.

## 4. Discussion

Treatment options are limited for MG patients who recur after standard chemoradiation. As oncology shifts towards precision medicine and personalized therapy, *EGFR* is an appealing therapeutic target in various cancer types due to its role in tumor growth and survival [3–6]. After the FLAURA trial reported practice changing outcomes with osimertinib in *EGFR*-mutant lung cancer, including patients with CNS involvement, osimertinib became an even more attractive agent to consider in patients with MGs with *EGFR* alterations [16].

Unlike lung cancer, gliomas contain a wide variety of *EGFR* alterations, which make it difficult to target. This study highlights the molecular heterogeneity of *EGFR* alterations in MG patients; the two most common *EGFR* alterations identified in this study were *EGFR* amplification and *EGFR vIII* mutation. *EGFR vIII* rearrangements are the most common EGFR rearrangement and are reported in 2457% of GBM [22, 23].

Two patients in our study with the *EGFR vIII* mutation remained on osimertinib for extended duration (236 and 294 days). Based on *in vitro* and *in vivo* studies, osimertinib has demonstrated preclinical activity in GBM with *EGFR vIII* rearrangements [9, 17, 24]. The extended period of treatment and disease stability of our *EGFR vIII* patients demonstrates the potential activity of osimertinib in select patients with *EGFR vIII* altered gliomas. Our study highlights the importance of identifying and characterizing *EGFR* alterations in MGs to determine in which patients treatment with osimertinib be considered.

Obtaining osimertinib for these patients is currently a challenge, as most insurance companies will not cover its costs due to its off-label use in MGs. Even under the best circumstances, obtaining an off-label drug may be quite time consuming. At WFBCCC, a unique precision medicine program is offered to match tumor genetics with potential novel treatments. In general, these results are used for later lines of therapy when standard therapy has been exhausted.

Tumor genetic information is obtained, ideally, from a new tissue sample after all prior lines of therapy, to better define the individual’s tumor and explore treatment options when necessary. This dedicated multi-disciplinary team expedites consent, outcome of benefit determination, and options for drug assistance and coverage that resulted in a median of 40 days from the time osimertinib was prescribed to receipt of the medication.

Although this study is limited in sample size, this heavily pretreated brain tumor population tolerated osimertinib therapy and half of the patients were able to achieve either PR or SD ranging from 77 to 294 days. To our knowledge, this is the largest study reporting on the clinical activity and tolerability of osimertinib in humans with MGs. Makhlin *et al* recently reported promising clinical activity with osimertinib in one patient with *EGFR*-mutant GBM [25]. Formal clinical trials are needed to evaluate osimertinib’s safety and efficacy in MGs as well as whether there is a benefit in combination with other therapeutic agents in the recurrent setting.

A future additional clinical trial consideration is whether osimertinib should be combined with temozolomide as a frontline, newly diagnosed treatment option in selected patients.

## 5. Conclusion

Overall, osimertinib may benefit select patients with recurrent MGs harboring *EGFR* alterations while having a tolerable safety profile.

Further clinical investigations are needed to assess the efficacy and safety of osimertinib in this brain tumor population, particularly in patients whose tumors express *EGFR vIII* rearrangements, and to identify which *EGFR* alterations may sensitize tumors to this BBB penetrant *EGFR-TKI*.

## Figures and Tables

**Table 1:** Genomic landscape of malignant gliomas treated with osimertinib.

Pt	*IDH1/2* Status	*EGFR* Alterations	Co-occurring Alterations	TMB (mut/Mb)	MSI Status
1	Wild type	(G598V)	*CDKN2A/B* homozygous del, *NF1* (P228fs*53), *PIK3R1* (L449fs*3), *MTAP* homozygous del, *pTERT* (-146C>T)	1	Stable
2	Wild type	Amplification, *vIII*, *vIVa*	*CDKN2A/B* homozygous del, *pTERT* (−124C>T)	5	Stable
3	Wild type	Amplification, *vIII*	*CDKN2A/B* homozygous del, *MTAP* homozygous del (exons 2–8), *pTERT* (−124C>T)	3	Stable
4	Wild type	Amplification, (R108K), (T263P), *vII, vIII*	*CDKN2A/B* homozygous del, *MTAP* homozygous del, *pTERT* (−124C>T)	3	Stable
5	Wild type	Amplification, *vIII*	*CDKN2A/B* homozygous del, *NF1* (K1661fs*36), *PTEN* truncation intron 7, *MTAP* homozygous del, *pTERT* (−124C>T)	3	Stable
6	Wild type	Amplification, (A289V), exon 20 insertion (D770_N771insSVD)	*CDKN2A/B* homozygous del, *PTEN* splice site (1027–2A>G), *SUFU* homozygous del (exons 11–12), *MLL2* (R2635Q), *pTERT* (−124C>T)	2	Stable

Abbreviations: Pt – patient, TMB – tumor mutational burden, MSI – microsatellite instability

**Table 2:** Baseline demographics and corresponding response and tolerability.

Pt	Age	Diagnosis	MGMT status	*EGFR* Alterations	# or recurrences	Regimens after RT + TMZ and adjuvant TMZ	KPS	Best response	Time to progression	Toxicity
1	74	GBM	Unmethylated	(G598V)	2	1. Clinical trial: AMG232 2. Bevacizumab Osimertinib + Bevacizumab	70	Unable to assess	−	Grade 3 thrombocytopenia
2	60	GBM	Methylated	Amplification, *vIII*, *vIVa*	1	1. *Repeat RT + TMZ 2. Bevacizumab 3. Osimertinib	50	SD	236 days	Grade 1 diarrhea, PNA
3	46	GBM	Methylated	Amplification, *vIII*	2	1. **TMZ 2. Osimertinib + TMZ (held for first month of osimertinib therapy) + Optune	90	PR	Still on treatment after 294 days	Grade 1 mucositis
4	53	Anaplastic Astrocytoma	N/A	Amplification, (R108K), (T263P), *vII, vIII*	2	1. Lomustine 2. Osimertinib	70- 80	PD	52 days	None
5	63	GBM	Unmethylated	Amplification, *vIII*	2	1. Bevacizumab 2. Osimertinib + Bevacizumab	70	Unable to assess	−	None
6	49	Anaplastic Astrocytoma	Unmethylated	Amplification, (A289V), exon 20 insertion (D770_N771insSVD)	2	1. Lomustine 2. Osimertinib	90	SD	77 days	Grade 2 thrombocytopenia

Abbreviations: Pt – patient, RT – radiation treatment, TMZ – temozolomide, KPS – Karnofsky performance status, GBM – glioblastoma, N/A – not available, SD – stable disease, PR-partial response, PD – progressive disease/refractory, PNA – pneumonia

* Enrolled on a clinical trial (ACT IV) for *EGFR vIII* rearrangement. Patient received a vaccine in combination with RT + TMZ and adjuvant TMZ.

** Received Optune and compassionate use pembrolizumab in combination with adjuvant TMZ for one year for PD-L1 mutation.
